# National ethics guidance in Sub-Saharan Africa on the collection and use of human biological specimens: a systematic review

**DOI:** 10.1186/s12910-016-0146-9

**Published:** 2016-10-22

**Authors:** Francis Barchi, Madison T. Little

**Affiliations:** 1 Edward J. Bloustein School of Planning and Public Policy, Rutgers University-New Brunswick, 33 Livingston Street, New Brunswick, NJ 08901 USA; 2 Department of Social Policy & Intervention, University of Oxford, Oxford, UK

**Keywords:** Sub-Saharan Africa, Biospecimens, Biobanks, Ethics guidance, Regulatory systems, Ethics committees, Health systems, Materials transfer agreements

## Abstract

**Background:**

Ethical and regulatory guidance on the collection and use of human biospecimens (HBS) for research forms an essential component of national health systems in Sub-Saharan Africa (SSA), where rapid advances in genetic- and genomic-based technologies are fueling clinical trials involving HBS and the establishment of large-scale biobanks.

**Methods:**

An extensive multi-level search for publicly available ethics regulatory guidance was conducted for each SSA country. A second review documented active trials listed in the WHO International Clinical Trials Registry Platform as of January 2015 in which HBS collection was specified in the protocol. Findings were combined to determine the extent to which countries that are study sites for HBS-related research are supported by regulatory guidance language on the collection, use, ownership and storage of biospecimens.

**Results:**

Of the 49 SSA countries, 29 had some form of national ethics guidance, yet only 17 provided language relating to HBS-related research, with specific guidance on consent (14), ownership (6), reuse (10), storage (9), and export/import/transfer (13). Ten countries accounted for 84 % of the active clinical trials involving the collection of HBS in SSA. All except one of these countries were found to have some national guidance in the form of regulations, codes of ethics, and/or standard operating procedures; however, only seven of the ten offered any language specific to HBS.

**Conclusions:**

Despite the fact that the bulk of registered clinical trials in SSA involving HBS, as well as existing and proposed sites for biorepositories under the H3Africa Initiative, are currently situated in countries with the most complete ethics and regulatory guidance, variability in the regulations themselves may create challenges for planned and future pan-African collaborations and may require legislative action at the national level to revise. Countries in SSA that still lack regulatory guidance on HBS will require extensive health system strengthening in ethics governance before they can be full participants in the modern research enterprise.

## Background

Exponential growth in the fields of pharmacogenetics and genomics research over the past decade is fueling worldwide interest in the establishment of biorepositories of human biological specimens. Biorepositories already exist in three SSA countries—The Gambia, South Africa, Zimbabwe [1–3]. Major expansion in this domain is occurring in Sub-Saharan Africa, largely driven by the Human Heredity and Health in Africa Initiative (H3Africa), a joint effort of the National Institutes of Health (NIH), the Wellcome Trust, and the African Society for Human Genetics [4]. The ‘hub and spoke’ model adopted by this initiative calls for the development of four pilot regional biobanks – two in South Africa and one each in Nigeria and Uganda – which will serve as multi-country repositories of specimens collected under sponsored research activities taking place throughout the region [5]. The H3Africa Initiative reflects significant efforts by the international community of researchers, funding agencies, and African academic institutions to build a foundation for sophisticated, state-of-the-art genomic research on burdensome diseases in Africa; the ultimate goal of the initiative is to improve the health of the African peoples.

From its inception, the initiative has sought broad consensus on topics relating to ethics and governance A Joint NIH and Wellcome Trust Policy document sets forth high-level principles on ethics, governance, and resource sharing with which research networks and programs funded by the initiative are expected to comply [6]. A standing working group on ethics and regulatory issues comprising ethicists and other members of the scientific community meets periodically to discuss and develop recommendations on a wide range of topics including community engagement strategies, cultural issues, informed consent, participant safety, return of results, intellectual property, material transfer agreements, and other regulatory issues. While such guidance has the potential to serve as a model framework for all countries in Africa to consider as they develop their own national guidelines, the current focus of the initiative is on those entities that it supports. Applied more broadly, the pan-continental approach espoused by H3Africa and similar initiatives may require a level of ethical guidance at the country level explicitly addressing the collection and use of HBS which is currently not to be found in most national ethics regulatory guidance. In many African countries, such systems are in their infancy; policies that address emerging technologies such as whole genome sequencing and association studies are absent [7, 8].

The International Declaration on Human Genetic Data, promulgated by UNESCO in 2003, recognized the importance of national policies to guide the collection, processing, use, and storage of human genetic data, as well as the review by local and institutional ethics committees of protocols involving such data [9]. In Africa specifically, a growing body of literature has focused on the need to have ethical and legal governance structures in place to oversee research involving HBS and the operation of biobanks [10, 11].

If the H3Africa vision of a pan-continental genomic enterprise is to be fully realized, ethics guidelines at the national level must align in ways that support interoperability among African countries. In cases where no guidelines presently exist, investment in institutional capacity-building by such entities as H3Africa and others may be needed to support their development. In cases where national guidelines do exist, some may require the addition of new language specific to research involving HBS; such revisions may require legislative approval. There may be instances where national values simply will not support the transnational transfer of genetic information, broad data sharing within research communities (however relevant to their populations), or the delegation of decision-making on future use to an outside authority. The lack of interoperability among African nations in their ethics guidance as well as differences in interpretation of meaning in instances where international recommendations have been adopted has the potential to slow, even derail, the realization of benefit from new advances and approaches in biomedical research [10, 12].

Although a number of scholars have examined the ethical, legal, and social issues associated with the collection and use of biospecimens in research, there has been far less focus on the existence and nature of regulatory guidance on HBS at the national level in Sub-Saharan Africa [8, 11–17]. Of particular importance is the identification of national ‘gaps’ in ethics guidance, as well as inconsistencies and national differences among those countries where guidance does exist.

## Methods

This study involved two systematic reviews designed to determine the availability of publicly accessible regulatory HBS guidance at the national level in SSA countries and to assess the extent to which it addressed ethical and regulatory issues relating to consent, ownership, reuse, storage, and export/import/transfer. Two reviews were conducted as part of this project. The first involved a multi-level search to identify publicly available national research ethics guidance on the collection and use of HBS in countries in Sub-Saharan Africa. This process, detailed in Table 1, involved keyword searches, on-line reviews of international ethics databases and regional and national government websites, and, in those instances where on-line efforts yielded no results, direct contact via email with national ethics committees and scans of ethics review statements included in previously published journal. National ethics guidance was operationalized to include enabling legislation for the creation and constitutions of national research institutes and national research ethics committees, codes of ethics, national ethics guidelines, and standard operating procedures. We excluded those documents that only provided instructions to researchers on how to submit their protocols, instruction on writing consent documents, and organizational information from research ethics committees that were not identified as being national in jurisdiction. Materials Transfer Agreements (MTA) and Specimen Transfer Agreements (STA) for specific countries were included in our search but were only used as a source of guidance if we could find a specific reference to “human” biological specimens. We limited our results to those national documents that we could access publicly on-line or secure through contact with the national regulatory body, reasoning that guidelines that were inaccessible via such mechanisms would be of limited utility to the research community.

**Table 1:** Search strategy and selection criteria

Level	Components of search
Level 1	Search Engines: Google, Google Scholar, PubMed, National Library of Medicine Keywords: Name of country + each of the following terms: human biological specimens, biobanks, ethics regulations, research ethics, ethics regulations, ethics committee, bioethics, consent, human subjects research, drug regulation, research regulations, institutional review boards, ethics review committees, materials transfer agreement
Level 2	On-line directories: Canadian Coalition for Global Health Research (CCGHR). www.ccghr.ca Harvard Research Ethics Guidelines International Online Navigation Map (REGION). https://www.hsph.harvard.edu/region-map/ Health Research Web (HRWeb). https://healthresearchweb.org/en/africa Office for Human Research Protections (OHRP). International Compilation of Human Research Protections. http://www.hhs.gov/ohrp/sites/default/files/internationalcomp2016%20.pdf Training and Resources in Research Ethics Evaluation (TRREE). http://elearning.trree.org/ UNESCO – Assisting Bioethics Committees (ABC) http://www.unesco.org/new/en/social-and-human-sciences/themes/bioethics/assisting-bioethics-committees/ UNESCO Global Ethics Observatory. http://www.unesco.org/new/en/social-and-human-sciences/themes/global-ethics-observatory/ WHO African Health Observatory (Health Systems) http://www.aho.afro.who.int/en/atlas/health-system WHO MINDbank. https://www.mindbank.info/
Level 3	Web sites of regional ethics organizations: Pan African Bioethics Initiative (PANBIN). http://www.who.int/sidcer/fora/pabin/en/
Level 4	Direct contact via email with National Ethics Committees in Sub-Saharan African countries
Level 5	1. Scan of journal articles on biomedical research in specific SSA countries to identify: a. Ethics Review Statement: Name of African REC, if any, that conducted an ethics review of research protocol b. Names of US researchers who have worked in that particular African country 2. Check web for identified IRB/ERC/approval granting regulatory body 3. Contact individual researchers by email to request information about IRB reviewing procedures and familiarity with regulations (if any) governing collection and use of HBS.

**Fig. 1:**
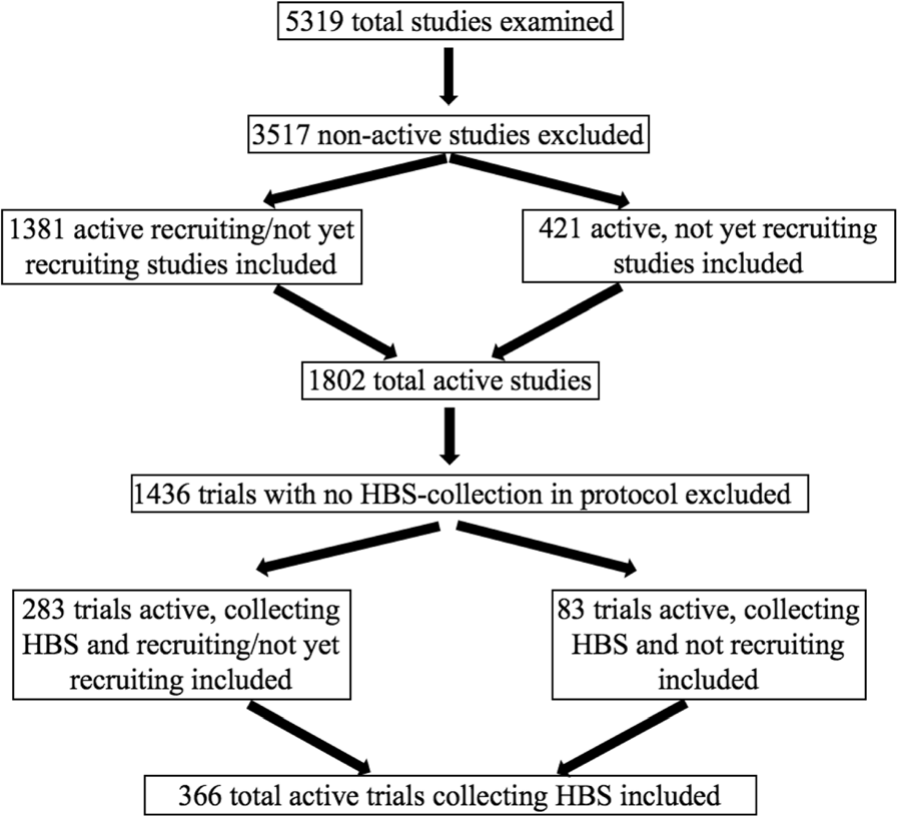
Research process for identifying active clinical trials in SSA involving HBS collection (Source: WHO-ICTRP as of January 1, 2015)

A second review was conducted using the WHO International Clinical Trials Registry Platform (ICTRP) to identify registered clinical trials taking place in Sub-Saharan Africa as of January 1, 2015, and, of these, the number of clinical trials in each country that called for the collection of HBS as part of the study protocol [18]. The ICTRP is a web-based, publicly accessible compilation of studies that have been registered in any of the following databases: Australian New Zealand Clinical Trials Registry (ANZCTR); Chinese Clinical Trial Registry (ChiCTR); ClinicalTrials.gov; EU Clinical Trials Register (EU-CTR); ISRCTN; The Netherlands National Trial Register (NTR); Brazilian Clinical Trials Registry (ReBec); Clinical Trials Registry – India (CTRI); Clinical Research Information Service - Republic of Korea (CRiS); Cuban Public Registry of Clinical Trials (RPCEC); German Clinical Trials Register (DRKS); Iranian Registry of Clinical Trials (IRCT); Japan Primary Registries Network (JPRN); Pan African Clinical Trial Registry (PACTR); Sri Lanka Clinical Trials Registry (SLCTR); and Thai Clinical Trials Register (TCTR). Included in the count of active clinical trials were those trials listed in the database as active/not recruiting; recruiting; not yet recruiting; authorized-recruitment/may be ongoing or finished; and pending. Studies were excluded if identified as not recruiting; complete; terminated; closed/follow-up continuing; closed to recruitment/follow-up complete; temporary halt or suspension; other; not applicable; or withdrawn. The process used to identify active trials calling for HBS collection is illustrated in Fig. 1.

## Results

Table 2 summarizes the availability of national ethics guidance irrespective of content as well as guidance language specific to HBS in each of the 49 countries included in the WHO region constituting Sub-Saharan Africa. Detailed information on the specific content of any HBS-related language is provided in Table 3. Twenty-nine (60 %) of the countries in the region had some national ethics guidance, either in the form of laws, regulations, codes, guidelines, or standard operating procedures. Of these 29 countries, however, twelve did not have regulations that addressed specific guiding principles or rules for ethics research or review. Six (Congo DRC, Equatorial Guinea, Republic of Guinea, Lesotho, Madagascar, and Mali) had regulatory language that was limited to establishing or defining the functions and constitution of research ethics committees. One (Mauritius) had drafted a code of research ethics that was distributed for public comment in 2003, but no documentation could be found that it had been operationalized. Three countries (Cameroon, Mozambique, Rwanda) offered limited language specific to guiding principles or ethical conduct of research, but link their review processes to the ethical guidance contained in the Declaration of Helsinki, CIOMS, or the ICH-GCP [19–21]. No national guidelines for general ethical conduct of research could be found for The Gambia, but the Medical Research Council – The Gambia had published very detailed guidelines for the collection and use of biospecimens stored in the National DNA Bank of The Gambia which we have included in our analysis [22]. In addition, two countries (Chadand Gabon) had been working with UNESCO on the establishment of national ethics committees but no regulatory documentation could be found [23, 24].

**Table 2:** SSA countries with HBS-related information in legislative/regulatory/guidance ethics documents

Country	Ethics system guidance (Gen’l)	Specific HBS language	Ethics system guidance specific to HBS					
			HBS informed consent	HBS ownership	HBS reuse	HBS storage	HBS export import transfer	HBS materials or specimens transfer agreement
Angola								
Benin	●	●						
Botswana	●	●	●	●	●	●	●	●
Burkina Faso	●	●	●					
Burundi								
Cameroon	●							
Cape Verde								
Central African Rep.								
Chad								
Comoros								
Rep. of Congo								
Congo (DRC)	●							
Cote d’Ivoire								
Djibouti								
Equatorial Guinea	●							
Eritrea								
Ethiopia	●	●					●	●
Gabon	●							
The Gambia	●	●	●	●	●	●	●	●
Ghana	●							
Rep. of Guinea	●							
Guinea- Bissau								
Kenya	●	●	●		●		●	●
Lesotho	●							
Liberia	●	●	●					●
Madagascar	●							
Malawi	●	●	●		●	●	●	●
Mali	●							
Mauritania								
Mauritius	●							
Mozambique	●						●	
Namibia								
Niger								
Nigeria	●	●	●			●	●	●
Rwanda	●	●	●	●			●	●
Sao Tome & Principe								
Senegal	●							
Seychelles								
Sierra Leone	●	●	●		●			●
Somalia								
South Africa	●	●	●		●	●	●	●
South Sudan								
Sudan	●	●	●		●			
Swaziland								
Tanzania	●	●	●	●	●	●		●
Togo								
Uganda	●	●	●	●	●	●	●	●
Zambia	●	●	●	●	●	●	●	●
Zimbabwe	●	●				●	●	●
TOTAL	29	17	14	6	10	9	12	13

**Table 3:** National regulatory guidance language on collection and use of HBS by country*

Legislation, regulations, and/or guidance re. research and/or HBS	Specific HBS language	HBS consent	HBS ownership	HBS secondary use	HBS storage	HBS Import/export
Angola						
None found	None found	None found	None found	None found	None found	None found
Benin						
Loi 2005-31 Portant prevention, prise en charge et contrôle du VIH/SIDA (2006) [28] Loi No. 2010-40 portant code d'éthique et de déontologie pour la recherche en santé en République du Bénin (8 December 2010) [29].	Loi 2005-31 portant prevention, prise en charge et conttrolé du VIH/SIDA en Republique du Benin (2005) Loi No. 2010-40 Portant code d'éthique et de déontologie pour la recherche en santé en République du Bénin (8 December 2010).	No language	No language	No language	No language	No language
Botswana						
Botswana Statute Law: Anthropological Research Act 1967 [30] Drugs and Related Substance Act, 1992 [31] Botswana Drugs Advisory Board: Guidelines on drug registration applications in Botswana [4th edition] 2014.[32] Botswana Ministry of Health,: Standard Operating Procedures for Review of Biomedical and Bio-behavioral Research in Botswana, 2011 [33]	Ministry of Health Standard Operating Procedures, Article 7.2 Procedures on Human Genetic Research, pages 103-108. Articles 8.1-8.5, Human Biological Materials, pages 111-114	Consent required unless waived by an ethics committee." (MOH SOPs, 2011, 7.2.iv) An ethics committee may sometimes waive, with or without conditions, the requirement for consent. (MOH SOPs, 2011, 7.2.v) Acquisition, storage and future use of biological materials: Informed consent process and forms separate from and in addition to informed consent for research participation. (MOH SOPs,2011, 8.2)	Government of Botswana is normally the owner of research data. The creators of the data, the Principal Investigators and local institutions in Botswana should hold the data in trust on behalf of the Botswana government and the research participant. The host institutions or researcher has to apply to the HRDC regarding the decisions about use and sharing of their data with other researchers and institutions according to Botswana law (MOH SOPs, 2011, 8.5.Appendix 11)."	Research protocols should detail the purpose and use of research specimens Where archived specimens are required in another experiment, researchers must provide a new protocol for review and approval by HRDC. (MOH SOPs, 2011, 8.2.iii)	Host institution in Botswana is entrusted custodianship of samples and should hold samples in trust on behalf of Botswana Government and the research participant. Research subjects have right to withdraw their samples if traceable. Health Research and Development Committee approval required for use, transfer, storage & future use according to Botswana laws. Where samples have not been obtained as part of research, the institution that collected them takes custodianship of them. Any future study on such samples is subject to review by HRDC. (MOH SOPs, 2011, 8.2)	No transfer unless: a) the researcher and the other research group are collaborating on research that has been approved by an ethics committee; and b) the genetic material cannot be identified. Transfer of identifiable or potentially identifiable genetic material may be approved by HRDC in certain circumstances. (MOH SOPS, 2011, 7.2.vi) All exchanges and transfers (including importation) of HBS for research purposes shall require clearance from the HRDC. (MOH SOPs, 2011, 8.3) Procedures for Exchange/Transfer of HB Materials, pg. 112:(MOH SOPs, 2011, 8.3)
Burkina Faso						
Law No. 23/94/ADP of 19 May 1994 on the Code of Public Health [34] Décret No. 2002-536 Established National Bioethics Committee [35] Minister of Health, the Minister of Secondary and Higher Education and Scientific Research (2004) Joint order No. 2004/147/MS/MESSRS on the organization and functioning of the Ethics Committee for Health Research in Burkina Faso. [36] (Decree contains no research ethics regulatory guidance.) A draft Code of Ethics was written in 2005 but there is no publicly available data indicating it was operationalized.	Human biological materials means the organs, tissues and products that can be used for therapeutic or research purposes on human beings. They can be derived from living or cadaveric donors. (Law No. 23/94)	Consent required for human genetic research unless waived by an ethics committee. Institutions or organizations wishing to conduct research on genetic material and on information collected for non-research purposes should develop and disseminate a general policy that informs subjects that such material and information may be used for future research, following research ethics committee approval, participants in such institutions or organizations should be informed that this policy exists and that their privacy and confidentiality will be protected. (p. 105-106). Page 106 specifies information that must be contained in a consent document for a study involving human genetic research. (Joint Order No. 2004)	No language	No language	No language	No language
Burundi						
None found	None found	None found	None found	None found	None found	None found
Cameroon						
Arrêté No. 079/A/MSP/DS- Order of the Minister of Public Health, October 22, 1987: Creation and organization of an ethics committee for research involving human subjects [37] (Article 9 refers to an evaluation process relying on Declaration of Helsinki) Decision 0674/D/MSP/CIRCB, October 13, 2006: Creation and functions of an ethics committee in the Chantal International Reference Center for research on the prevention and treatment of HIV/AIDS [38]	No language	No language	No language	No language	No language	No language
Cape Verde						
None found	None found	None found	None found	None found	None found	None found
Central African Republic						
None found	None found	None found	None found	None found	None found	None found
Chad						
Part of UNESCO Bioethics project. Efforts to establish National Ethics Commission reported in meeting report, UNESCO, 2008) [23]	None found	None found	None found	None found	None found	None found
Comoros						
None found	None found	None found	None found	None found	None found	None found
Republic of Congo						
None found	None found	None found	None found	None found	None found	None found
Congo (DRC)						
Ministry of Health, Politique nationale de recherché sure les systems de santé en RDC (June 2004) [39]	No language	No language	No language	No language	No language	No language
Côte D’ivoire						
None found	None found	None found	None found	None found	None found	None found
Djibouti						
None found	None found	None found	None found	None found	None found	None found
Equatorial Guinea						
Presidential Decree No. 124/2014: Establishes the National Ethics Committee of Equatorial Guinea (CENGE), 2014. [40] Presidential Decree 125/2014: Constitute CENGE and appoints inaugural members, 2014 [41]	No language	No language	No language	No language	No language	No language
Eritrea						
None found	None found	None found	None found	None found	None found	None found
Ethiopia						
FDRE Ministry of Science & Technology; National Research Ethics Review Guideline [5th Ed.] Sept. 2014 [42]	National Health Research Ethics Review Guideline, Guideline 8.3.2, p. 51-52	IC process should include info on HBS and data to be collected, data anticipated to be derived from sample as well as health and other records to be accessed, their intended uses, storage and duration of storage. Participants must be informed that specific information will not be shared with family members, and that, if shared with third parties, will be anonymized. Participant should be given option of allowing sharing of samples or not. (Guideline, 8.3.2., p. 52) Separate consent form for HBS collected for storage and/or future use. Participant may choose whether to have HBS stored for future studies. (Guideline, 9.2, p. 57)	Host institution in Ethiopia holds HBS in trust on behalf of research participant. (Guideline, 9-2, p. 58) Where HBS have not been obtained as part of research, the institution that collected the samples takes custodianship. (Guideline 9.2, p. 58)	Any shared samples must be anonymized to the recipient, Use of HBS beyond what is stated in original protocol must have consent of research participants or their representatives. Secondary use may only be done on anonymized samples and after getting approval by the IRB. When subsequent use of HBS or data is proposed that is not consistent with original I/C, a new consent should be obtained from participant/guardian or LAR or a waiver of consent should be requested from IRB. (Guideline, 8.3.2, p. 52)	Intent to store HBS and place and duration of storage must be specified in initial consent document (Guideline, 8.3.2) Research participants reserve right to withdraw their HBS from storage if samples are linked. (Guideline, 9.2)	MTA required. It must contain details regarding purpose of transfer/export, arrangements for collaboration and benefit-sharing, a framework for accessing and sharing data, restrictions to third party transfers, and annual reports to host institution. An Ethiopian scientist must be included as a co-investigator in all future studies using the HBS. The IRB in Ethiopia must review all research studies on stored HBS. (Guideline 9.2, p. 58)
Gabon						
Establishment of a National Research Ethics Committee (UNESCO 2007) [24]	No language	No language	No language	No language	No language	No language
The Gambia (Republic oF)						
[No national regulatory guidance for general research although final drafts exist.] Dept. of State for Health & Welfare; National Health Research Policy[revised draft for discussion: 29/10/2009] [43] Dept. of State for Health & Welfare; National Health Research Strategic Plan 2010-2014 [final draft: 15/01/2010] [44]	Guidelines for The Gambia National DNA Bank, 2001 [22]	Samples from children only after permission from parents or guardians No consent for reuse, but permission of Gambian government/MRC Laboratories Ethics Committee is required. Permission from donor for reuse of samples that have not been anonymized is required in principle, but committee can waive this requirement. (DNA Bank Guidelines, 2001)	DNA Collections are jointly owned by the Gambian people through their government representatives and the Medical Research Council (DNA Bank Guidelines, 2001)	Reuse permissible with approval from Joint Committee. Different levels of permissions/consents required depending on whether or not requested specimens have been anonymized, collected as part of a specific protocol or available to researchers with approval from Committee only. [DNA Bank Guidelines, 2001]	The Gambia National DNA Bank was established in 2001, the first biobank on the African continent (DNA Bank Guidelines, 2001).	Permission for export based on signed agreement between researchers and the DNA Bank, with approval of Committee. Agreement must describe analyses to be undertaken with specimens; feedback must be given to MRC Labs, results of analyses must eventually be placed in the public domain, and any manuscripts resulting from research must be submitted to Joint Committee prior to submission. (DNA Bank Guidelines, 2001)
Ghana						
No research ethics regulatory guidance Public Health Act 2012 [Act 851] Good Clinical Practice Doc No FDA/SMC/CTD/GL- GCP/2013/02 Ver. 2, 18 Dec 2015 (Adopted 27 Jan 2016) [45]	No language	No language	No language	No language	No language	No language
Guinea (Republic of)						
Decree No. D/218/PRG/SGG: On the Establishment, Functions and Organization of the National Ethics Committee for Research in Health (CNERS) (Decree No D/218). 1998. [46] Decree contains no ethics regulatory guidance language.	No language	No language	No language	No language	No language	No language
Guinea-Bissau						
None found	None found	None found	None found	None found	None found	None found
Kenya						
The Science, Technology and Innovation Act, 2013 [47] National Council for Science and Technology Guidelines for Ethical Conduct of Biomedical Research Involving Human Subjects in Kenya (NCST No. 45) 2004. [48] Kenya Medical Research Institute (KEMRI), National Ethics Review Committee: Guidelines and Standard Operating Procedures, 2004. [49] Ministry of Health, National Guidelines for Research and Development of HIV/AIDS Vaccines, 2005. [50] Pharmacy and Poisons Board: Guidelines for applications to conduct clinical trials in Kenya, 2011. [50]	HBS reference limited to Guideline 15 (National Guidelines, 2004)	Guideline 15. Informed Consent for epidemiological studies- ERC to determine whether or not individual informed consent is needed for studies involving "left-over" HBS (Natl Guidelines 2004) Applications for clinical trials must include patient information leaflets and informed consent forms for any proposed archiving of biological specimens for later research or genetics research. (Pharmacy & Poisons Board Guidelines, 2011, Art. 4.12)	No language	Guideline 15. Informed Consent for epidemiological studies- ERC to determine whether or not individual informed consent is needed for studies involving "left-over" HBS (Natl Guidelines 2004)	No language	No biological material transfer is permitted without the informed consent of the trial participants and without approval of protocol and in accordance to Ministry of Health guidelines on transfer of HBS. (Vaccine guideline 7.3, p. 44 2005) MTAs govern all transferred materials and specimens used for vaccine studies. MTAs must state that specimens will only be used for scientific, educational, non- commercial use. Any other uses require a cooperative research and development agreement (RADA). (Vaccine Guidelines, 8.3, 2005)
Lesotho						
Ministry of Health and Social Welfare: National Health and Social Welfare Research Policy (NHSWRP), Lesotho. 2007.[51] (Established a National Health and Social Welfare Research Ethics and Clearance Committee within the National Research Institute at the MoHSW.) No research ethics regulatory guidance.	No language	No language	No language	No language	No language	No language
Liberia						
Ethics approval required from one of three REC bodies: Liberia Institute of Biomedical Research/National Health Science Research EC (LIBR/NHSREC), the University of Liberia Institutional Review Board (UL- IRB), or the newly established National Research Ethics Board (NREB). [52] University of Liberia-Pacific Institute for Research and Evaluation: Institutional Review Board (IRB) Policies and Procedures Handbook 2008 [53] Liberia Medicines and Health Products Regulatory Authority, Guideline for Application to Conduct Clinical Trials in Liberia 2014. [54]	Research involving the collection or study of pathological specimens, or diagnostic specimens may be exempt, if those sources are publicly available or if the information is recorded by the investigator in such a manner that the subjects cannot be identified, directly or indirectly, through identifiers linked to the subjects. Archival research in which individual subjects could potentially be identified is not exempt. (University of Liberia, Policies & Procedures Handbook, 2008, Art. 5 pg. 23)	Applications for clinical trials must include patient information leaflets and informed consent forms for any proposed archiving of biological specimens for later research or genetics research. (LMHRA Guidelines, 2014, Art. 4.12.5 p. 14)	No language	No language	No language	Materials Transfer Agreement must be provided to the Liberia Medicines and Health Products Regulatory Authority’s (LMHRA) (LMHRA Guidelines, 2014.)
Madagascar						
Decree No. 5855/99-SAN (June 17, 1999) modified by Decree No. 4583/2000-SAN (May 8, 2000) (Created a National Ethical Committee for Biomedical Research.) Documents contain no research ethics regulatory guidance. [55]	No language	No language	No language	No language	No language	No language
Malawi						
National Health Sciences Research Committee: General Guidelines on Health Research (December 2007) [56] National Health Sciences Research Committee, Policy Requirements, Procedures, and Guidelines for the Conduct and Review of Human Genetic Research in Malawi (September 2012) [23] National Commission for Science and Technology (2012). National Policy measures and Requirements for the Improvement of Health Research Coordination in Malawi (Revised ed., 11/2012). [57]	Clear explanation and justification for the collection and exporting of biological samples will have to be made. (General Guidelines, 2007, Section 6.1) HBS language contained in 2007 and 2012 documents.	Informed consent for HBS required. Persons may only be consented for HBS collection for purposes of answering the study objectives of a presently intended study that has been clearly defined.(Policy Requirements, 2007, 10.0)	No language	All forms of studies & testing aimed at collecting and storing HBS for future unspecified genetic research/analysis, including scientific retrospective analysis is non- permissible (HBS Guidelines, 2012, 3.4.7) Plans, attempts, requests for obtaining HBS for future research non-permissible (HBS Guidelines, 2012, 3.4.8)	Procedures for specimen storage will have to be clearly defined. (General Guidelines, 2007, Section 6.1) Not permissible to consent participants to collection, use, storage of specimens for future use, HBS may be stored for future analysis as specified in a presently intended study approved by NSTC but not for a period to exceed five years. Additional time period may be granted. (Policy Requirements, 2007, 10.0)	Analysis of specimens should be done within Malawi by local technicians/professionals. Export permitted only in exceptional circumstances when the needed technology does not exist in Malawi nor can it be imported or when tests are needed to confirm results and/or quality control and validation are required.(Policy Requirements, 2007, 10.0) MTAs are required. Agreement must clearly specify why export is necessary, its intended use, length of time HBS will be kept, and name of local technician/professional who will be responsible for HBS testing.(Policy Requirements, 2007, 10.0)
Mali						
No. 02-200/P-RM April 22, 2002- Creation of a National Ethics Committee for Health and Life Sciences [58] Minister of Health- Internal Ruling August 26, 2004- Operation and Functions of Ethics Committee for Health and Life Sciences. [59] Loi 86-11 N RM Fundamental Principles of Scientific and Technological Research [60] (Documents contain no ethics guidance for conduct of research.) Organization and functions of the National Institute of Public Health Research, including establishment of an ethics committee within Institute) [61]	No language	No language	No language	No language	No language	No language
Mauritania						
None found	None found	None found	None found	None found	None found	None found
Mauritius						
The Clinical Trials Bill (Feb 11, 2010)- calls for Ethics Committee to uphold International Ethics provisions while respecting customs and values of country. [62] Mauritius Research Council (MRC) drafted ethics guidelines for biomedical research involving human subjects in July 2003 and they remain out for public comment. No on-line evidence that final guidelines have been approved.	No language	No language	No language	No language	No language	No language
Mozambique						
Order May 21, 2002- Minister of health established National Research Ethics Committee [63] No specific laws on research involving human beings. National ERC (CNBS-Comité national de Bioética para Saude) applies international principles (Dec. of Helsinki, CIOMS, ICH-GCP.)	No language	No language	No language	No language	No language	No language
Namibia						
Ministry of Health and Social Sciences has constituted a Biomedical Research Ethical Committee but it is not operational. [64]	None found	None found	None found	None found	None found	None found
Niger						
None found	None found	None found	None found	None found	None found	None found
Nigeria						
National Health Research Ethics Committee of Nigeria (NHREC) National Code of Health Research Ethics 2007. [65] Policy Statement on Storage of Human Samples in Biobanks and Biorepositories in Nigeria (PS1.02013) (November 1, 2013) [66]	Reference to HBS in connection with MTAs (Ethics Code, 2007) See Biobanks (Biobank Policy, 2013) NHREC to provide sole oversight of ethical aspects of biobanking (Biobank Policy, 2013)	Biobanks in Nigeria must retain copies of all consent forms and these must be matched to all samples in biobanks (Section D,iii,a., Biobanks, 2013) NHREC supports Broad Consent- consent in which the type or purpose of research is defined in broad terms and for a work that is not specified by time (Section E.1, Biobanks, 2013)	No language	No language	Any samples kept more than 2 months post-analysis are considered 'banked' and covered by Biobank Policy. Copies of all participant I/C forms must be available and can be matched to samples stored in biobanks (Biobank Policy 2013)	Export permitted, subject to MTA approved by HREC. Institutional HRECs shall grant final approval for research involving international transfer of HBS. (HREC Code, 2007)
Rwanda						
Rwanda Ministry of Health, National Research Ethics Committee: Standard Operating Procedures, 2009 [67] Rwanda Ministry of Health, National Health Research Committee: Operational Guidelines, 2012. [68]	Regulations with respect to HBS (Sections 35 & 36 of Rwanda Ministry of Health Standard Operating Procedures, 2009.	HBS must be collected with free and informed consent even if tissue is obtained as part of patient care. Consent forms must indicate if HBS is being collected for current research only, how long the specimens will be kept, and when they will be destroyed. If HBS is stored longer than the current research, then a separate permission for storage must be obtained.	Under MTAs: Ownership is the "Provider of the samples." Ownership of any income resulting from commercialization must be negotiated in good faith (Rwanda SOPs, 2009, Appendix 7).	No language	No language	MTAs are required. Entities to whom HBS is transferred must provide any resultant publications, and provider must be part of the publication team. (Rwanda SOPs, 2009, Appendix 7)
Sao Tome & Principe						
None found	None found	None found	None found	None found	None found	None found
Senegal						
Arrêté ministerial No. 3224 MSP- DERF-DER, March 17, 2004: Creation and organization of National Council for Health Research (CNRS) [69] Loi No. 2009-17, March 9, 2009: Code of Ethics for Health Research. [70] Règlement Intérieur du Conseil National de la Recherche en Santé [Standard Operating Procedures], March 7, 2006. [71]	No language	No language	No language	No language	No language	No language
Seychelles						
None found	None found	None found	None found	None found	None found	None found
Sierra Leone						
Pharmacy Board of Sierra Leone: Guidelines for Conducting Clinical Trials of Medicines, Food Supplements, Vaccines, and Medical Devices in Sierra Leone (Version 02) (G-SLClinTrial). 2014. [72] Pharmacy Board of Sierra Leone: Guideline for Good Clinical Practice (GCP) in Sierra Leone (Version 01) (SL-GCPs). 2014 [73 73] Ministry of Health & Sanitation, Office of the Sierra Leone Ethics and Scientific Review Committee: Sierra Leone Ethics and Scientific Review Committee –Guidelines. n.d.[74]	Section 3.9 Biological specimens/samples (Guidelines for conducting clinical trials, 2014, p. 20-21) Definition of HBS: a biological specimen or a biological sample is defined as material derived from various animal and human sources (e.g., blood, tissues, and cells) used to treat and prevent diseases. (Guidelines for GCP, 2014)	Separate consent for use of HBS (Guidelines, 2014, Sec. 3.9.1) HBS taken in course of clinical care may be used for research without consent subject to ethics committee review. Patients have right to know their HBS is being used for research. Patient refusal to such use does not warrant waiver of consent. Refusals to be honored except in case of public health emergency. (Guidelines, 2014, Sec. 3.9.2)	No language	Secondary use of HBS is constrained by conditions specified in initial consent. Therefore, initial consent should specify whether or not there will be secondary use; conditions under which subjects must be re-contacted; plans, if any, for de-identification; and subject rights. (Guidelines, 2014, Sec. 3.9.3)	No language	Materials Transfer Agreement is required. (NIAID Communication with the Pharmacy Board of Sierra Leone (PBSL) (October 2014–January 2015)
Somalia						
None found	None found	None found	None found	None found	None found	None found
South Africa						
Act 61 of 2003 National Health Act [75] Regulations Relating to the Use of Human Biological Material, March 2012 [76]	Chapter 8, Sections 53-68. (National Health Act, 2003) Regulations Relating to the Use of HBS (2012)	Written informed consent of individual with provisions for consent from subjects who are minors or mentally ill (Regulations, 2012, 3.1) Written informed consent by users/donors for release of stored information and for long-term storage of genetic material, stem cells, or research findings (Regulations, 2012, 13.d & f).	No language	HBS information used for purposes for which it was originally intended. (Regulations, 2012, 13.e).	Written informed consent of the user or donor for long term storage of genetic material, stem cells, or research findings. HBS information treated confidentially. Users' written informed consent prior to release of stored information. (Regulations 2012, 13e & f).	Export permit is required. No export unless it is established that sample was donated under terms of Act and will be used in accordance with terms of Act. (Act, 2003, 8.68.1(g))
South Sudan						
None found	None found	None found	None found	None found	None found	None found
SUDAN						
National Guidelines for Ethical Conduct of Research Involving Human Subjects (2008) [77]	Guidelines, 2008, Secs. 5.2, 5.10, & 5.11).	Investigator must provide subjects: policy to use results of genetic tests and familial genetic information and precautions to prevent disclosure to others (Guidelines, 2008, 5.2.16); possible research sites, direct or secondary use of HBS taken in course of clinical care (5.2.18); disposal, storage, future use of HBS (5.2.19); any commercial products from HBS and distribution of any revenues (Guidelines, 2008, 5.2.20) Consent forms must have separate sections requesting use of HBS for research purposes (Guidelines, 2008, 5.10) HBS taken in course of clinical care may be used w/o consent subject to approval of ethics committee (Guidelines, 2008, 5.11).	No language	Constrained by conditions specified in original consent (Guidelines, 2008, 5.12)	No language	No language
Swaziland						
None found	None found	None found	None found	None found	None found	None found
TANZANIA						
National Institute for Medical Research Act, 1979 [78] Guidelines of Ethics for Health Research in Tanzania, 2nd ed., 2009. [24] The Human DNA Regulations Act, 2009 [79] Standard Operating Procedures for the National Health Research Ethics Review Committee, 2007 [80]	Part V Human DNA Research Activities, Medical Research & Treatment (Act, 2009).	Consent for intended study only. New protocol required for reuse (Guidelines, 2009, 8.8) No collection of a sample without the consent of the sample source (DNA Act, 2009, 38-41).	Samples of Human DNA are the property of the sample source. (DNA Act, 2009, 28.2.b) Where the research results from an individual or an institute are new or unique, the researcher or the institute shall have the intellectual property rights (DNA Act, 2009, 5.3.7).	Consent for intended study only New protocol required for reuse (8.8, Guidelines, 2009) Sample destroyed on completion of analysis unless sample source's representative has previously directed otherwise in writing (DNA Act, 2009, 28.2.e) After completion of original research, investigators must (a) within one month notify of their intent to reuse and secure the permission of sample source or (b) destroy samples within three months (38.44, DNA Act, 2009, 38.44)	No language	Materials transfer agreement is required.
Togo						
None found	None found	None found	None found	None found	None found	None found
Uganda						
Uganda Ministry of Health, National Health Policy, 1999 [81] Uganda National Council for Science and Technology (NCST), National Guidelines for Research Involving Humans as Research Participants, July 2014. [82]	See Guidelines 2014, Sec. 10., pp. 28-31.	Consent must include explanation of how HBS will be managed at end of study. If stored, separate consent must be obtained, with purpose of study, quantities stored, location, measures to ensure confidentiality, future governance, risks and potential benefits. (Guideline, 2014, 5.3.h)	Sample sources own the samples If samples are identified, source may withdraw them at any time. Samples to be held in trust by duly authorized Uganda organization. Trustee organization has authority to decide use, transfer, storage and future use of HBS in its care, taking into account rights and welfare of research participants (Guidelines, 2014, 10.3) If MTA doesn't specify ownership of future products, they are automatically owned by provider organization. (Guidelines 2014, 10.5)	A Ugandan scientist must be included on all future studies. Separate informed consent document required for each reuse (See HBS, I/C). All future studies subject to REC review. If samples collected initially for purposes other than research, sample sources must be traced and consent for research use secured. (Guidelines,2014, 10.2)	See HBS I/C	MTA required. Future use of HBS subject to review and approval by an REC in provider's country. (Guideline, 2014, 10.4) Applicant for transfer must be a legal resident of Uganda and affiliated with a locally registered and recognized Uganda organization. (Guidelines, 2014, 10.5)To export, requesting entity must demonstrate lack of in-country capacity to perform tasks. Sample may be transferred for quality assurance and laboratory reference purposes. All exchanges and transfers require approval of NCST except exchanges within country. Capacity-building by requesting organization encouraged. (Guidelines, 2014, 10.4)
Zambia						
The National Health Research Act 2013 [83]	May only be collected for purposes stated in research protocol (Act, Part VI) Minister of Health may designate specific sites as biobanks	No HBS removed from living person for health research purposes without written consent of donor in accordance with provisions of the Act. Act, Art. 47 (1)	Ownership specified in MTA as determined by Minister of health in consult with Health Authority. Act, Art. 51 (1)(a)	A person shall not withdraw HBS from a living person for any unspecified future health research activity of unspecified storage. Act, Art. 47 (2)	A person shall not withdraw HBS from a living person for any unspecified future health research activity or unspecified storage. Act, Art. 47 (2) Storage not to exceed 10 years without special approval of longer period from Health Authority Act, Art. 51 (2) (b)	Only with written approval of the National Health Research Authority Act, Art. 50 (1) and if terms of MTA are met Art. 50 (2) MTA is required. Minister, in consultation with Authority, decides on requirements.
Zimbabwe						
Scientific Technological Research Act (Research Act) (Ch. 10:22), 1986 (Replaced Research Act 1959) [84] Medical Research Council of Zimbabwe: Ethics Guidelines for Health Research Involving Human participants in Zimbabwe, Version 1.4, September 30, 2011 [85] Research Council of Zimbabwe (RCZ): Specimens Transfer Agreement (STA) (online as of 1/3/15) [86]	Discussion of HBS not included in MRCZ guidelines.	No language	No language	No language	Extraterritorial storage of biospecimens beyond Research Council of Zimbabwe approval period is illegal. RCZ STA, 2015) Note: All international studies must be registered with RCZ.	Export for specified research purposes only. No third party transfers. Approval of REC required. Specimens Transfer Agreement (STA) is required.

* Guidance language cited verbatim where possible.

**Fig. 2:**
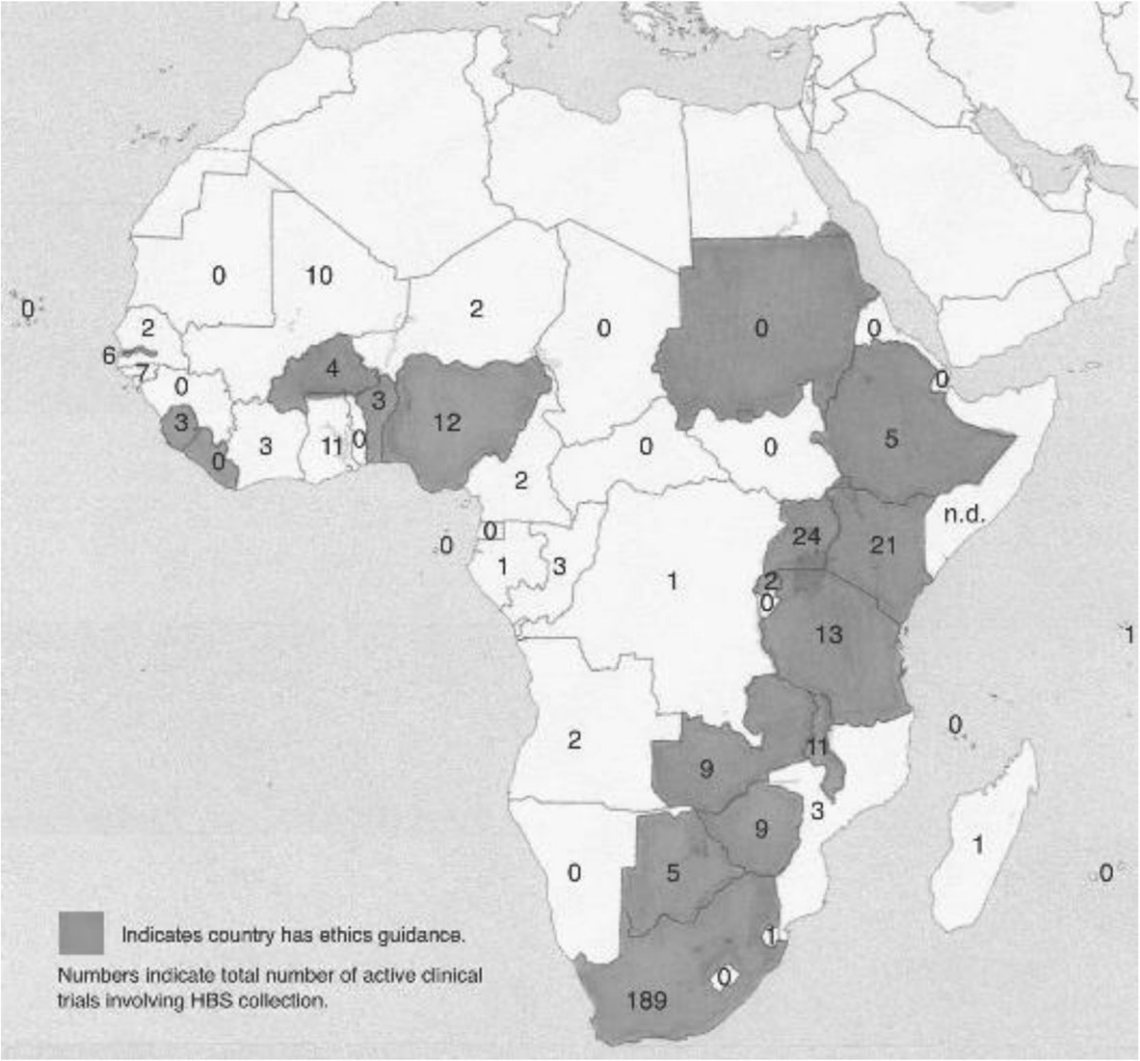
Distribution of active clinical trials in SSA involving HBS collection (Trial information Source: WHO-ICTRP as of January 1, 2015; Map source: Wikimedia Commons)

More than one half (59 %) of the countries whose guidelines articulated ethical principles or guidelines for ethical review include some language to guide the collection and use of HBS for biomedical research. Fourteen of these offered some language with respect to informed consent; 10 addressed the subject of reuse; and 12 contained specific language relating to export/import/transfer. Only six countries offered language with respect to HBS ownership and nine contained language regarding storage. Thirteen required that a Materials or Specimens Transfer Agreement be executed. Table 4 reports the findings with respect to registered, active clinical trials in Sub-Saharan Africa that called for the collection of HBS. Of the 5319 studies identified in the WHO International Clinical Trials Registry Platform, 1802 of were currently active as of January 1, 2015. Three hundred sixty-six (366) of these involved the collection of a biosample from research subjects. The distribution of these trials across the region are illustrated in Fig. 2.

**Table 4:** Clinical trials in SSA involving HBS collection (Source: WHO-ICTRP as of January 1, 2015)

Country	Total # studies	Total active recruiting/Not yet recruiting	Total active not recruiting	Total active studies	HBS collection active recruiting/Not yet recruiting	HBS collection active not recruiting	Total trials collecting HBS
Angola	3	2	0	2	2	0	2
Benin	33	10	1	11	2	1	3
Botswana	50	10	5	15	4	1	5
Burkina Faso	117	20	11	31	3	1	4
Burundi	0	0	0	0	0	0	0
Cameroon	57	12	6	18	1	1	2
Cape Verde	0	0	0	0	0	0	0
Central African Rep.	6	5	3	8	0	0	0
Chad	0	0	0	0	0	0	0
Comoros	0	0	0	0	0	0	0
Republic of Congo	59	10	3	13	3	0	3
Congo (DRC)	17	10	2	12	1	0	1
Cote d’Ivoire	37	10	4	14	3	0	3
Djibouti	0	0	0	0	0	0	0
Equatorial Guinea	0	0	0	0	0	0	0
Eritrea	1	0	0	0	0	0	0
Ethiopia	74	22	7	29	2	3	5
Gabon	34	5	0	5	1	0	1
The Gambia	79	11	4	15	5	1	6
Ghana	138	33	7	40	10	1	11
Guinea	74	1	2	3	0	0	0
Guinea-Bissau	43	5	9	14	2	5	7
Kenya	320	65	27	92	17	4	21
Lesotho	0	0	0	0	0	0	0
Liberia	7	1	1	2	0	0	0
Madagascar	12	1	0	1	1	0	1
Malawi	171	33	15	48	7	4	11
Mali	101	20	7	27	7	3	10
Mauritania	5	3	0	3	0	0	0
Mauritius	9	2	1	3	0	0	0
Mozambique	52	11	1	12	2	1	3
Namibia	0	0	0	0	0	0	0
Niger	19	7	1	8	2	0	2
Nigeria	103	37	7	44	10	2	12
Rwanda	57	8	1	9	2	0	2
Sao Tome & Principe	0	0	0	0	0	0	0
Senegal	58	10	3	13	2	0	2
Seychelles	2	0	1	1	0	1	1
Sierra Leone	11	3	3	6	1	2	3
Somalia	n.d.	n.d.	n.d.	n.d.	n.d.	n.d.	n.d.
South Africa	2712	832	234	1066	150	39	189
South Sudan	0	0	0	0	0	0	0
Sudan	0	0	0	0	0	0	0
Swaziland	7	4	0	4	1	0	1
Tanzania	263	45	14	59	10	3	13
Togo	8	5	0	5	0	0	0
Uganda	351	74	25	99	20	4	24
Zambia	147	31	11	42	5	4	9
Zimbabwe	82	23	5	28	7	2	9
Total	5319	1381	421	1802	283	83	366

No data (n.d.)

Table 5 reports on the ethics system guidance on HBS found in the countries where the largest number of studies involving HBS are currently taking place in Sub-Saharan Africa. These 10 countries account for nearly 82 % of the active clinical trials involving the collection of HBS in the region that were registered in the WHO International Clinical Trials Registry Platform as of January 1, 2015. They included the five countries that either have existing biorepositories (The Gambia, South Africa and Zimbabwe) or planned to establish regional biorepositories in the near future as part of the H3Africa Initiative (Nigeria, South Africa and Uganda). All ten of these countries had some form of ethics system guidance in the form of regulations, codes of ethics, standard operating procedures, or guidelines, and, with the exception of Mali, provided some regulatory language at the national level as to the collection and use of HBS. Notably only four of these countries (The Gambia, Kenya, Uganda, and Zambia) included language in their national ethics documents on the ownership of HBS. Mali, where at the time of our study there were currently 10 registered active trials requiring a biosample, had no guidance language on HBS in its national ethics documents. Although most of the countries in which large biorepositories existed or are planned as part of H3-Africa contain fairly detailed HBS language in their guidelines, only two (The Gambia and Uganda) spoke to issues of ownership. After Mali, Zimbabwe offered the least guidance within this group of countries, with no language on HBS-specific consent, ownership, or reuse.

**Table 5:** National ethics guidance on 10 countries host to the most registered clinical trials

Country	# Active studies w/HBS	Existing biobanks	H3Africa regional biobanks	Ethics system guidance (Gen’l)	Ethics system HBS language	Ethics system guidance specific to HBS					
						Consent	Ownership	Reuse	Storage	Export	MTA
South Africa	189	●	●●	●	●	●		●	●	●	●
Uganda	24		●	●	●	●	●	●	●	●	●
Kenya	21			●	●	●		●		●	
Tanzania	13			●	●	●	●	●	●		●
Nigeria	12		●	●	●	●			●	●	●
The Gambia	11	●		●	●	●	●	●	●	●	●
Malawi	11			●	●	●		●	●	●	●
Mali	10			●							
Zambia	9			●	●	●	●	●	●	●	●
Zimbabwe	9	●		●	●				●	●	●
Total	309	3	4	10	9	8	4	7	8	8	8

## Discussion

This study examined the availability of national ethics and regulatory guidance on biomedical research in Sub-Saharan African countries and identified the extent to which national regulatory systems provided ethics guidance on specific aspects of HBS collection and use to inform research design and ethics review. Ethical principles and regulatory guidance regarding HBS consent, ownership, reuse, storage, and export/import/transfer are foundational elements in national health systems as research activities calling for the collection and use of biospecimens in Africa expand and ethics committees are increasingly called on to review and respond to rapidly advancing innovation in research and research technologies.

Our research found that despite efforts at the international, regional, and national levels, 20 of the countries in Sub-Saharan Africa (41 %) at the time of our study still lacked articulated national ethical principles and regulatory guidance for policy-making, review, and monitoring of research. Among those countries where ethical and regulatory guidance existed, specific language with respect to HBS collection and use was often lacking or incomplete. While in 17 of the countries in Sub-Saharan Africa (59 %), some form of national regulatory guidance relating to HBS did exist, a number of these lacked explicit guidance on HBS-related issues such as consent (3), ownership (11), reuse (7), storage (8), and export/import/transfer (5).

Gaps in national ethical and regulatory guidance in the region on the collection and use of HBS for research may result in inconsistent reviews within ethics committees and pose challenges for full participation by African countries in genomic research. In the absence of guiding principles and regulatory direction, ethics committees may delay or deny HBS-related research of potential benefit to their communities, or they may approve such research without due exploration of its implications for its citizenry or its cultural values.

Differences in ethical and regulatory requirements among African countries where guidance does exist also appear to raise impediments to the interoperable pan-African approach to genomic research espoused by H3Africa. Despite the fact that the bulk of registered clinical trials involving HBS in Sub-Saharan Africa, as well as existing sites and those sites proposed for future biorepositories, are currently situated in countries with the most complete ethics and regulatory guidance on HBS, variability in the regulations themselves may create challenges for pan-African collaborations that rely on transnational sharing of specimens and data or that delegate decision-making responsibility with respect to future use to repository-centric ethics or scientific review committees. Malawi, for example, contained language in its guidelines that all forms of studies and testing aimed at collecting and storing HBS for future unspecified genetic research/analysis is ‘non-permissible’ [25]. Tanzania required that a new consent be secured for each new research protocol in which a sample will be reused [26]. Uganda, where one of the H3Africa repositories is intended to be located, required that all requests for transfers of HBS be made by a legal resident of Uganda who was affiliated with a locally registered and recognized Ugandan organization [27].

While these and other potential sources of regulatory tension may be resolved in the future, for now they represent potential constraints on research that calls for a more ‘regional’ perspective of specimen and data sharing, and are likely to require legislative action at the national level to revise. At the same time, gaps in HBS-related guidance within countries that are already research-intensive sites may give rise to lengthy or inconsistent review decisions by local ethics committees left to make their own policy decisions in the absence of clearly stated regulations or principles at the national level.

Future growth in the fields of pharmacogenetics and genomic research is likely to generate demand for HBS from a more representative pool of the African peoples and to prompt researchers to look for new communities in countries where research is currently not active. The transnational movements of diseases themselves, as seen in the 2014 Ebola outbreak in Western Africa, may identify new target populations for study beyond those of interest at present. Such expansion will require system-wide capacity-building in these settings and will need to be guided by clearly articulated national regulatory guidance to ensure ethical and thoughtful research governance.

Resources to assist national governments in developing ethics capacity may be available through such programs as UNESCO’s Assisting Bioethics Committees (ABC) program, which aims to build and strengthen bioethics infrastructure within its member states and which is already working to build capacity in Chad, Gabon, and Madagascar [23, 24, 28]. H3Africa’s working group on ethics and regulatory issues and its work to date in articulating broad principles and regulatory guidance regarding HBS collection and use [6] would, were its focus to extend beyond its own funded research networks and programs, be a valuable starting point for countries as they begin to build national consensus on the value of genomics research for their communities and the region as a whole.

### Limitations of this study

Data used in this study were largely derived from publicly accessible sources. Despite an extensive search, some of the countries in Sub-Saharan Africa that we report as lacking national ethics regulatory guidelines may, in fact, have them. Given the central role that national policies play in shaping the research landscape in the region, it is important that researchers and ethics committees have ready access to country-level guidance on the collection and use of HBS as the scope of genomics research in Sub-Saharan Africa expands. In addition, the mapping of active clinical trials that involve HBS in Sub-Saharan Africa relied on publicly accessible international registries. It therefore did not take into account those studies which are not required to register, and serves more as an illustration of a potential regulatory ‘gap’ between research and national governance than as a comprehensive source of data on HBS-related research in the region.

## Conclusion

Despite substantial capacity-building efforts, many countries still lack regulatory guidance on the collection and use of human biological specimens in research. Although the African countries currently most in demand as HBS-related clinical research sites number among those offering the most guidance, extensive health system strengthening will be needed to ensure that regulatory language is available to guide the ethical extension of HBS-related research into other countries in the region. Efforts to create interoperability across national policies to meet international and regional research goals of Pan-African research collaboration must be on-going, recognizing that some differences may be value-based choices by representative governments while others, although malleable to change, will require legislative action to amend.

## References

[1] Sirugo G, van der Loeff S, Sam O, Nyan O, Pinder M, Hill AV (2004). A national DNA bank in The Gambia, West Africa, and genomic research in developing countries. Nat Genet.

[2] Matimba A, Oluka MN, Ebeshi BU, Sayi J, Bolaji OO, Guantai AI (2008). Establishment of a biobank and pharmacogenetics database of African populations. Eur J Hum Genet.

[3] University of Kwazulu-Natal African Center for Health and Populations Studies http://www.africacentre.ac.za/index.php/data-rep.

[4] Human Heredity and Health in Africa (H3Africa) http://www.h3africa.org/. Accessed 10 Oct 2015.

[5] Bruinenberg M, Frey M, Napier M, Summers A (2014). Comparing the hub-and-spoke model practices of the LifeLines study in the Netherlands and the H3Africa Initiative. Biopreserv Biobank.

[6] H3Africa. Terms of reference for the Ethics and Regulatory Issues Working Group. http://h3africa.org/consortium/working-groups/24-working-group-external-pages/128-tor-ethics-wg-exp. Accessed 10 Oct 2015.

[7] Nyika A, Kilama W, Chilengi R, Tangwa G, Tindana P, Ndebele P, Ikingura J (2009). Composition, training needs and independence of ethics review committees across Africa: are the gate-keepers rising to the merging challenges?. J Med Ethics.

[8] Wright GEB, Koornhof PGB, Adeyemo AA, Tiffin N (2013). Ethical and legal implications of whole genome and whole exome sequencing in African populations. BMC Med Ethics.

[9] UNESCO (2003). International declaration on human genetic data.

[10] Barchi F, Matlhagela K, Jones N, Kebaabetswe PM, Merz JF (2015). “The keeping is the problem”: a qualitative study of IRB-member perspectives in Botswana on the collection, use, and storage of human biological samples for research. BMC Med Ethics.

[11] Staunton C, Moodley K (2013). Challenges in biobank governance in Sub-Saharan Africa. BMC Med Ethics.

[12] Dove ES, Tasse A-M, Knoppers BM (2014). What are some of the ELSI challenges of international collaborations involving biobanks, global sample collection, and genomic data sharing and how should they be addressed?. Biopreserve Biobank.

[13] Akintola SA (2013). Ethical and legal issues in biobanking for genomic research in Nigeria. BEOnline.

[14] Andana PA (2008). Human tissue-related inventions: ownership and intellectual property rights in international collaborative research in developing countries. J Med Ethics.

[15] Muula AS, Mfutso-Bengo JM (2007). Responsibilities and obligations of using human research specimens transported across national boundaries. J Med Ethics.

[16] Sathar MA, Dhai A (2012). Laws, regulations and guidelines of developed countries, developing countries in Africa, and BRICS regions pertaining to the use of human biological material (HBM) in research. S Afr J BL.

[17] Sathar MA, Dhai A, van de Linde S (2014). Collaborative international research: ethical and regulatory issues pertaining to human biological materials at a South African institutional research ethics review committee. Dev World Bioethics.

[18] World Health Organization. International Clinical Trials Registry Platform. http://apps.who.int/trialsearch/Default.aspx. Accessed 10 Oct 2015.

[19] World Medical Association. Declaration of Helsinki - Ethical Principles for Medical Research Involving Human Subjects [64th WMA General Assembly, October 2013]. http://www.wma.net/en/30publications/10policies/b3/. Accessed 10 Oct 2015.

[20] Council for International Organizations of Medical Sciences (CIOMS). International Ethical Guidelines for Biomedical Research Involving Human Subjects 2002. http://www.recerca.uab.es/ceeah/docs/CIOMS.pdf. 14983848

[21] The International Conference on Harmonization of Technical Requirements (ICH). E6 Good Clinical Practice: Consolidated Guidance. 1996. http://www.fda.gov/downloads/Drugs/Guidances/ucm073122.pdf.

[22] Medical Research Council (MRC) –The Gambia. Guidelines of the National DNA Bank, The Gambia. Fajara, The Gambia: Medical Research Council, 2001.

[23] UNESCO: Meeting to discuss the establishment of the National Bioethics Committee, N'Djamena, Chad, 29 July - 2 August, 2008. [SHS/EST/ABC/REP/10]. unesdoc.unesco.org/images/0016/001627/162750E.pdf.

[24] UNESCO: 1st Preparatory Meeting on the Establishment of a National Ethics Committee in Gabon [SHS/EST/ABC/REP/09/Rev1]. 2007. http://unesdoc.unesco.org/images/0015/001559/155952e.pdf.

[25] Malawi, National Health Sciences Research Committee. Policy Requirements Procedures and Guidelines for the Conduct and Review of Human Genetic Research in Malawi. 2012. Section 3.4.1 http://www.medcol.mw/comrec/wp-content/uploads/2014/07/Human_Genetic_Research_Procedures_and_Guidelines.pdf.

[26] Tanzania National Health Research Forum. Guidelines of Ethics for Health Research in Tanzania, 2nd Ed. [published by National Health Research Ethics Committee]. 2009; Section 8.8 http://clinregs.niaid.nih.gov/documents/tanzania/G-EthicsHR.pdf.

[27] Republic of Uganda, Uganda National Council for Science and Technology. National Guidelines for Research involving Humans as Research Participants. July 2014. https://www.swarthmore.edu/sites/default/files/assets/documents/institutional-review-board/Human_Subjects_Protection_Guidelines_July_2014.pdf.

[28] UNESCO: Meeting with the Malgasch National Commission for the Ethics of Science and Technology (CMEST), Antananarivo, Madagascar, 20–25 June, 2007. http://unesdoc.unesco.org/images/0015/001528/152831E.pdf.

[29] République du Benin. Loi No. 2005–31 du 05 Avril 2006: Portant prevention, prise en charge et contrôle du VIH SIDA en République du Benin. 2006. http://www.ilo.org/wcmsp5/groups/public/---ed_protect/---protrav/---ilo_aids/documents/legaldocument/wcms_125247.pdf.

[30] République du Benin (2010). Loi No. 2010–40 du 08 Decembre 2010: Portant code d’éthique et de déontologie pour la recherché en santé en République du Benin.

[31] Republic of Botswana. The Anthropological Research Act. 1967. http://www.elaws.gov.bw/docs/statutes/Botswana%20Statute%20Law%201967.pdf.

[32] Republic of Botswana (1992). Drugs and related substances [Act No. 18 of 1992].

[33] Republic of Botswana, Drug Advisory Board (2014). Guidelines on drug registration applications in Botswana.

[34] Republic of Botswana, Ministry of Health (2011). Standard operating procedures for review of biomedical and bio-behavioral research.

[35] Burkina Faso: Loi No. 23/94/ADP of 19 May 1994 in the Code of Public Health, 1994.

[36] Burkina Faso: Decret No. 2002-536/Portant creation d’un Comité d’éthique pour la recherché en santé au Burkina Faso, 2002.

[37] Burkina Faso: Arrêté conjoint 2004/147/MS/MESSRS. Portant organization et functionnement du Comité d’éthique pour la recherche en santé au Burkina Faso (CERS), 2004.

[38] Republic of Cameroon, Ministry of Public Health (1987). Arrêté No. 079/A/MSP/DS du Octobre 1987. Pourtant création et organization d’un comité d’éthique sur la recherché impliquant les êtres humains.

[39] Republic of Cameroon, Ministry of Public Health (2006). Décision No. 0674/D/MSP/CIRCB du 13 Octobre 2006.

[40] Democratic Republic of Congo, Ministry of Health (2004). Politique nationale de recherché sur les systems de santé en République Democratique du Congo.

[41] Committee of Equatorial New Guinea (CENGE). 2014. http://guineaecuatorialpress.com/noticia.php?id=5849&forcedfoto=1414523929.jpg&lang=en. Accessed 10 Oct 2015.

[42] Republic of Equatorial Guinea. Decree No. 125/2014 to constitute and name the inaugural members of the CENGE. 2014. http://guineaecuatorialpress.com/noticia.php?id=5849&forcedfoto=1414523929.jpg&lang=en.

[43] Ethiopia (FDRE), Ministry of Science and Technology (2014). National Research Ethics Guideline.

[44] The Gambia, Department of State for Health & Social Welfare. National Health Research Policy: Research to protect and improve health, 2010–2014 [Revised draft for discussion 29/12/09].

[45] The Gambia, Department of State for Health & Social Welfare. National health Research Strategic Plan: Research to protect and improve health, 2010–2014 [Final draft: 15 January 2010]

[46] Food and Drugs Authority (FDA). Guidelines for good clinical practice in Ghana. FDA/SMC/CTD/GL-GCP/2013/2. 27 January 2016.

[47] Republic of Guinea: Decree No. D/218/PRG/SGG. On the Establishment, Functions and Organization of the National Ethics Committee for Research in Health (CNERS). 1998. http://cners-guinee.org/wp-content/uploads/2014/02/Decret-.pdf.

[48] Republic of Kenya. Acts 2013: Special Issue. Republic of Kenya: Kenya Gazette Supplement No. 43 (Acts. No. 28) 25 Jan 2013.

[49] Kenya, National Council for Science and Technology. Guidelines for ethical conduct of biomedical research involving human subjects in Kenya (NCST No. 45). 2004. https://www.nacosti.go.ke/newsletter/doc_download/69-guidelines-for-ethical-conduct-of-research-involving-human-subjects-in-kenya.

[50] Kenya Medical Research Institute (KEMRI) (2004). National Ethics Review Committee Guidelines and Standard Operating Procedures (SOPs).

[51] Republic of Kenya, Ministry of Health (2005). Kenya National Guidelines for Research and Development of HIV/AIDS Vaccines.

[52] Republic of Kenya, Pharmacy and Poisons Board (2011). Guidelines for applications to conduct clinical trials in Kenya.

[53] Kingdom of Lesotho (2007). National Health and Social Welfare Research Policy (NHSWRP), Lesotho.

[54] Liberia Institute of Biomedical Research (2011). Liberia Institute of Biomedical Research/National Health Science Research Ethics Committee Guidelines: procedures for researchers.

[55] University of Liberia-Pacific Institute for Research and Evaluation (2008). Institutional Review Board (IRB) policies and procedures handbook.

[56] Republic of Liberia, Liberia Medicines and Health Products Regulatory Authority (2014). Guideline for application to conduct clinical trials in Liberia.

[57] Committee MNHSR (2007). General guidelines on health research.

[58] Malawi: National Commission for Science and Technology (2012). National policy measures and requirements for the improvement of health research co-ordination in Malawi [Sections 18 & 48 of the Science and Technology Act of 2003].

[59] Republic of Mali (2002). Creation of a National Ethics Committee for Health and Life Sciences, No. 02-496/P-RM.

[60] Republic of Mali (2004). Minister of Health – Internal ruling: operation and functions of Committee for Health and Life Sciences.

[61] Republic of Mali (1986). Fundamental principles of scientific and technological research, Loi 86–11 N RM.

[62] Republic of Mali (2006). Organization and functions of the National Institute of Public Health Research, 06–301 P-RM.

[63] Republic of Mauritius (2010). The clinical trials bill (No. XIX of 2010).

[64] Republic of Mozambique, Ministry of Health (2002). Order of Minister of Health establishing the Comité national de bioética para saude.

[65] World Health Organization (WHO). African Health Observatory (AFRO): Namibia- Research. http://www.aho.afro.who.int/profiles_information/index.php/Namibia:Research_-_Health_information,_research,_evidence_and_knowledge. Accessed 10 Oct 2015.

[66] Nigeria, Federal Ministry of Health, National Health Research Ethics Committee of Nigeria (NHREC) (2006). National code of health research ethics.

[67] Nigeria, Federal National Health Research Ethics Committee of Nigeria (2013). Policy statement on storage of human samples in biobanks and biorepositories in Nigeria (PS1.02013).

[68] Republic of Rwanda, Ministry of Health, National Ethics Committee (2009). Standard operating procedures.

[69] Republic of Rwanda, Ministry of Health (2012). Guidelines for researchers intending to do health research in Rwanda.

[70] Republic of Senegal. Arrêté ministerial No. 3224 MSP-DERF-DER [March 17, 2004]. http://www.jo.gouv.sn/pip.php?article5037.

[71] Republic of Senegal. Loi No. 2009–17, Code of Ethics for Health Research [March 9, 2009]. http://elearning.trree.org/mod/folder/view.php?id=179.

[72] Republic of Senegal. Règlement Intérieur du Conseil National de la Recherche en Santé (Standard Operating Procedures) [March 7, 2006].

[73] Republic of Sierra Leone, Ministry of Health and Sanitation, Pharmacy Board of Sierra Leone (2014). Guidelines for conducting clinical trials of medicines, food supplements, vaccines, and medical devices in Sierra Leone (Version 02) (G-SLClinTrial).

[74] Republic of Sierra Leone, Ministry of Health and Sanitation, Pharmacy Board of Sierra Leone (2014). Guideline for Good Clinical Practice (GCP) in Sierra Leone (Version 01) (SL-GCPs).

[75] Republic of Sierra Leone, Ministry of Health & Sanitation, Office of the Sierra Leone Ethics and Scientific Review Committee. Sierra Leone Ethics and Scientific Review Committee –Guidelines. n.d.

[76] Republic of South Africa. Act 61 of 2003 National Health Act, September 2003. [Current version in force]. http://www.lawsofsouthafrica.up.ac.za/index.php/current-legislation.

[77] Republic of South Africa. Regulations relating to the use of human biological material [Government Notice R177 in Government Gazette 35099]. March 2, 2012. http://www.lawsofsouthafrica.up.ac.za/index.php/browse/medical-and-health/national-health-act-61-of-2003/regulations-and-notices/61-of-2003-national-health-act-regs-gnr-183-2-mar-2012-to-date-pdf/detail.

[78] Republic of the Sudan, National Ministry of Health (2008). National guidelines for ethical conduct of research involving human subjects.

[79] United Republic of Tanzania (1979). National Institute for Medical Research Act, No. 23 of 801979.

[80] United Republic of Tanzania (2009). The Human DNA Regulation Act.

[81] United Republic of Tanzania, National Institute of Medical Research (2007). Standard Operating Procedures for the National Health Research Ethics Review Committee.

[82] Republic of Uganda, Ministry of Health (1999). National health policy.

[83] Government of Zambia (2013). National Health Research Act, No. 2 of 2013.

[84] Republic of Zimbabwe (1986). Research Act, Chap 10:22.

[85] Republic of Zimbabwe, Medical Research Council of Zimbabwe. Ethics Guidelines for Health Research involving Human Participants in Zimbabwe, Version 1.4. [September 30, 2011] https://www.daidscrss.com/partners/Page_Regulatory_Management/Docs_Regulatory_Management/Docs_Flag_Forms/Zimbabwe_conducting_health_research_in_zim.pdf.

[86] Republic of Zimbabwe, Medicines Control Authority of Zimbabwe. Guidelines for good clinical trial practice in Zimbabwe. 2012. http://www.mcaz.co.zw/index.php/downloads/category/11-guidelines.

